# Antireflection Surfaces for Biological Analysis Using Laser Desorption Ionization Mass Spectrometry

**DOI:** 10.1155/2018/5439729

**Published:** 2018-10-31

**Authors:** Jing Yang, Hongjun Zhang, Jia Jia, Xinrong Zhang, Xiaoxiao Ma, Minlin Zhong, Zheng Ouyang

**Affiliations:** ^1^State Key Laboratory of Precision Measurement Technology and Instruments, Department of Precision Instrument, Tsinghua University, Beijing 100084, China; ^2^School of Materials Science and Engineering, Tsinghua University, Beijing 100084, China; ^3^College of Materials and Chemistry & Chemical Engineering, Chengdu University of Technology, Chengdu 610000, China; ^4^Department of Chemistry, Tsinghua University, Beijing 100084, China

## Abstract

Laser desorption ionization mass spectrometry (LDI-MS) is a primary tool for biological analysis. Its success relies on the use of chemical matrices that facilitate soft desorption and ionization of the biomolecules, which, however, also limits its application for metabolomics study due to the chemical interference by the matrix compounds. The requirement for sample pretreatment is also undesirable for direct sampling analysis or tissue imaging. In this study, antireflection (AR) metal surfaces were investigated as sample substrates for matrix-free LDI-MS. They were prepared through ultrafast laser processing, with high light-to-heat energy conversion efficiency. The morphology and micro/nanostructures on the metal surfaces could be adjusted and optimized by tuning the laser fabrication process. The super-high UV absorption at 97% enabled highly efficient thermal desorption and ionization of analytes. The analytical performance for the matrix-free LDI was explored by analyzing a variety of biological compounds, including carbohydrates, drugs, metabolites, and amino acids. Its applicability for direct analysis of complex biological samples was also demonstrated by direct analysis of metabolites in yeast cells.

## 1. Introduction

Matrix-assisted laser desorption ionization (MALDI), along with electrospray ionization (ESI), is one of the two most successful ionization techniques for the mass spectrometry (MS) analysis of biomolecules. Before the use of chemical matrices, laser desorption ionization (LDI) of biological compounds had suffered high fragmentation of analytes and low efficiency of desorbing intact molecules, which limited its application for analyzing biomolecules of relatively large molecular weights [1]. The chemical matrix compounds used in MALDI efficiently absorb the laser energy and transfer the heat for desorption [2, 3], so softer ionization [4, 5] could be achieved for a wide range of analytes, such as peptides, proteins, and polymers [6–9]. MALDI, however, also has some known issues. The assisting matrix compounds themselves are easily ionized during the LDI process, which produces extensive chemical noises. This makes MALDI not suitable for analysis of metabolites in the mass range of <500 Da. The inhomogeneous matrix cocrystallization with the analytes leads to poor reproducibility for analysis, which significantly impacts the quantitation precision. The chemical treatment of samples also is not desirable for direct analysis applications such as tissue imaging [10, 11].

Research efforts have been devoted to explore various materials, e.g., nanomaterials, as the LDI substrates in replacement of chemical matrices. In 1999, the desorption/ionization on silicon (DIOS) method [12] was developed, using porous silicon as the substrate for direct and soft LDI. The advantageous features of DIOS included little background noises in the low-mass range and high sensitivity of detection. With the rapid development in material science and engineering, nanomaterials with porous structures have been studied as the substrates or matrices for LDI, mainly including silicon-based substrates [13, 14], carbon-based substrates [15–17], metal [18, 19], and metal-oxide matrices [20]. Various functionalized materials were investigated for LDI-MS for analyzing biomolecules in the mass range below 500 Da, with little chemical interferences [21, 22].

Matrix-free LDI using functional surface represents an attractive approach for MS analysis [23, 24] since the sample preparation can be significantly simplified and alteration to sample surface could be avoided. The desorption process in LDI is mostly dominated by thermal desorption due to the rapid, laser-induced temperature increase of substrate during the short pulse of laser irradiation, enhanced by some other nonthermal processes [25–28]. It was suggested that in matrix-free LDI the efficiency depended on the UV absorption and laser-induced temperature rise capacity of the substrates, as well as the interaction between the analytes and the substrate. These are influenced by the surface morphology and surface functionalization [25, 29, 30]. Since all events in desorption and ionization are driven by the laser pulse energy, the light-to-heat energy conversion capacity of the substrate is important for matrix-free LDI [31].

In this study, we have investigated a novel approach for performing matrix-free LDI-MS analysis using antireflection (AR) metal surfaces [32] as the sample substrates. The AR surfaces were fabricated using one-step direct writing with ultrafast laser, which produced quasi-periodical arrays of protrusions covered with nanofeatures. Broad band light adsorption, e.g., over 90% in average and as high as 97% for UV region, could be achieved [33]. The high photothermal conversion efficiency is ideal for thermal desorption process through rapid heating of the microstructures. In comparison with other chemically modified materials, the AR surfaces are extremely durable and reusable. The LDI-MS performance with the AR substrates was investigated through analysis of a variety of chemical and biological compounds, including carbohydrates, amino acids, and drugs. Efficient and soft LDI was achieved for MS analysis, with minimal chemical interferences and molecular fragmentations leading to good ionization efficiency and analysis sensitivity. This method was also applied for analyzing real-world samples, with oligosaccharides in the onion extract and metabolites in yeast cells successfully detected.

## 2. Results

### 2.1. Micro/Nanofeatures of the AR Surface

**Figure 1:**
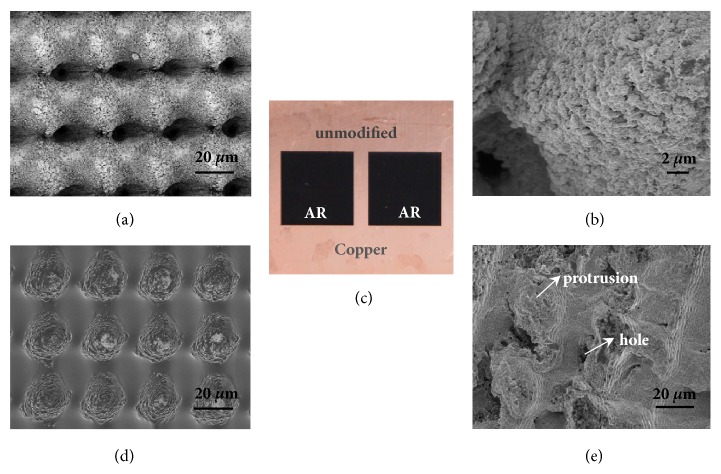
**Characterization of the AR surface**. SEM micrographs for (a) micropatterns on surface and (b) nanoscale features on surface of protrusions and holes. (c) AR surface fabricated from copper. (d) AR surface after sample deposition. (e) AR surface after multiple laser irradiation (~10^6^ laser shots on 1 cm^2^ substrate).

The AR metal surfaces were fabricated using femtosecond laser pulses. By simply adjusting the laser parameters and scanning factors, diverse micro/nanointegrated structures could be fabricated, with different impacts on the light absorption. For example, the depth of microholes depended on the scanning speed, the periodicity of microholes depended on the scanning interval of laser beam, and the size of microholes depended on the diameter of the laser spot. The femtosecond laser pulses produced hybrid micro/nanostructures on the metal surface.  Figure 1(a) shows the scanning electron microscope (SEM) micrograph of the uniformly distributed microarrays with a hole-to-hole distance of 35* μ*m, and the magnified micrograph in Figure 1(b) shows the nanoscale features on the surface of protrusions and holes. The super-high light absorption was achieved by geometrical trapping light with multiple reflections inside the microstructures. This process was accompanied by the excitation of surface plasmons and surface plasmon resonance (SPR) with the nanostructures [32]. For Cu nanoparticles with a radius from 1.7 to 6 nm, absorption peaks vary between 593 and 607 nm due to SPR. The aggregation of Cu nanoparticles can lead to a broadening effect of the resonance bands [34]. Further chemical treatments could also be used to fabricate nanowires, enhancing the absorption effect and altering the surface chemical properties. Due to the high absorptivity in the visible region, the Cu surface turns to be pitch black (Figure 1(c)).

The large surface area of the AR surface contributes to retaining analyte and solvent molecules [35]. The reflectance of the AR surfaces (prepared at 40mm/s) was measured as a function of wavelength using a UV-VIS-NIR spectrophotometer(UV-3600, Shimadzu Corp., Tokyo, Japan). A comparison has also been made for the surface before and after an intense use (Supporting Information, [Sec supplementary-material-1]). The AR surface absorbs the incident laser light efficiently and is applicable for a wide spectral range. It can serve as a very effective energy converting medium. The walls of the holes get rapidly superheated due to the limited heat dissipation inside the quasi-one-dimensional holes, thus facilitating the explosive evaporation of adsorbates.  Figure 1(d) shows the surface after depositing 1* u*L aqueous extract of onions and drying in the air. SEM image indicates that the microholes were partially but evenly filled with the sample solution. For use as LDI substrates, the AR surfaces have some distinct advantages. The microstructure of the AR surface is well controllable. In comparison with other LDI substrates with chemically produced nanofeatures, the AR surfaces are very stable and can be stored in atmosphere for months. Although oxidation might occur, it would not affect the surface morphology and light absorption capacity. The AR surface can also be reused for many times, simply with proper ultrasonic cleaning in methanol and water after each use. No cross contamination was observed when the AR material was reused for analyzing different types of analytes and no significant loss in sensitivity was observed either (Supporting Information, [Sec supplementary-material-1]-[Sec supplementary-material-1]). The cleaning process does not cause any damage to the surface nanostructures. Technically, each MS spectrum was obtained by averaging spectra over 500 laser shots and each sample spot was measured by 5~10 times. Usually, 1 cm^2^ of the AR surface can accommodate 200~400 sample spots, each with a diameter of 1~2 mm.

Generally, the pulse energy required for laser desorption is much lower than that used in laser processing; thus, the laser irradiation during LDI theoretically would not affect the microstructures of the metal surface. However, after prolonged and multiple laser irradiations, the microstructures of the surface could be inevitably damaged. In our experiments, typically five independent replicates were performed on each sample, and each MS spectrum was obtained by averaging over 500 laser shots.  Figure 1(e) shows the SEM micrograph of the AR surface structure after it was used for LDI, approximately with a total of 10^6^ laser shots on an area of 1 cm^2^. Each pulse energy was between 50 to 100* μ*J. Compared with Figure 1(a), the microstructures became irregular after large number of irradiations, but still with protrusions and holes. As a result, the light absorption and desorption efficiency would be reduced accordingly.

### 2.2. Hydrophobicity of the AR Surface

The hydrophobicity of the AR surfaces was found to be variable and had a significant impact on the surface-sample interaction. As mentioned above, changes in laser scanning parameters will lead to variations in the micro/nanostructures produced. We compared AR surfaces fabricated at different scanning speeds, by keeping other parameters constant (see Materials and Methods). The hole depth increased as the scanning speed decreased, resulting in more efficient light trapping inside the microstructures and therefore enhanced light absorption. They were used as LDI substrates and compared for MS analysis of several molecules. It was found that the surface prepared at 40 mm/s showed improved signal quality for most analytes tested for LDI-MS. The contact angles of different surfaces were measured to be 130°to 150°, a strong evidence suggesting a hydrophobic property of the AR surface.

Previous studies have shown that after ultrafast laser micro/nanostructuring, metal alloys initially exhibited super-hydrophilic behavior but became hydrophobic over time [36, 37]. For polished copper, the surface exhibited hydrophilicity due to the high surface free energy [38]. It first exhibited enhanced hydrophilicity right after femtosecond laser processing, due to enhanced surface roughness. Exposed in air for several hours, the structured surface became hydrophobic and super-hydrophobic eventually, with no changes in the structure itself. Detailed chemical analysis of the surface attributed the hydrophobicity to the adsorption of organic matters by the surface from the surrounding atmosphere [38]. The microstructures could also trap air, preventing the penetration of hydrophilic solvent into them [37, 39, 40]. When the surface was immersed into water, the surface cannot be immediately wetted and a layer of air could be seen. Following ultrasonic treatment, air bubbles collapse and the microholes could then be filled with water. The surface then turned super-hydrophilic and a water droplet could spread completely. After exposure to air for a longer time, the surface turned back to be hydrophobic, and the contact angle increased with the exposure time. The change in contact angle was measured as a function of time for exposure to air as shown in [Sec supplementary-material-1] (Supporting Information). The time required to regain hydrophobicity increased with the depth of the microholes, which generally takes about 40 minutes for the surface prepared at 40 mm/s and longer time to stabilize.

**Figure 2:**
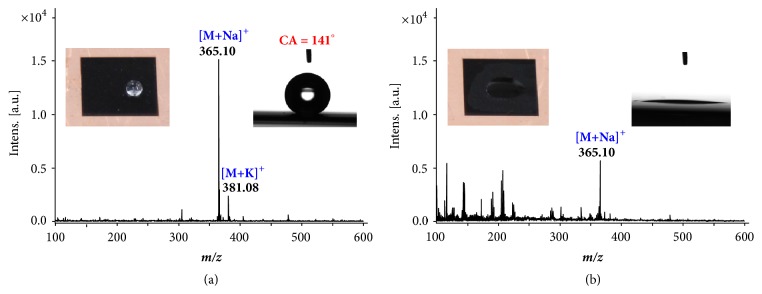
**Comparison of the AR surface in different wetting states.** Mass spectra recorded with LDI-MS for lactose (1 nmol) using the same AR surface under (a) hydrophobic and (b) hydrophilic state. The insets show a sample droplet on the AR surface and the corresponding static contact angles (CA).

This cycle can be repeated many times, changing the surface to be either hydrophobic or hydrophilic. For LDI-MS analysis, the surface hydrophobicity was foreseen to have an impact on the sample preparation. Analysis of lactose (MW 342.30) was used for a comparison with the same AR substrate (scanning speed: 40 mm/s) but in hydrophobic (Figure 2(a)) or hydrophilic (Figure 2(b)) conditions. Lactose solution of 2* μ*L (1* m*M) was directly dropped on the AR surfaces, dried in air and then analyzed using LDI-MS. Intensity for the same quantity of lactose was observed to be higher under the hydrophobic surface condition. For sample preparation, the aqueous sample droplet was confined in a circular area with a diameter of 1 to 2 mm on the hydrophobic surface but spread on the hydrophilic surface (see Figure 2 insets), which caused a significant difference in the local concentration of the analytes on surface. It can be concluded that hydrophobic surfaces offer better MS responses for the same quantity of aqueous samples. This finding is consistent with results previously reported in other studies [12]. Among the five surfaces prepared at different scanning speeds, the one prepared at 40 mm/s produced with moderately high scanning speed had good hydrophobicity, high light absorption and short wettability conversion cycle; it was then identified as the best substrate for practical LDI applications. The surfaces fabricated at 40 mm/s in the hydrophobic condition were then used for all experiments in this study.

### 2.3. Matrix-Free LDI with AR Substrates

**Figure 3:**
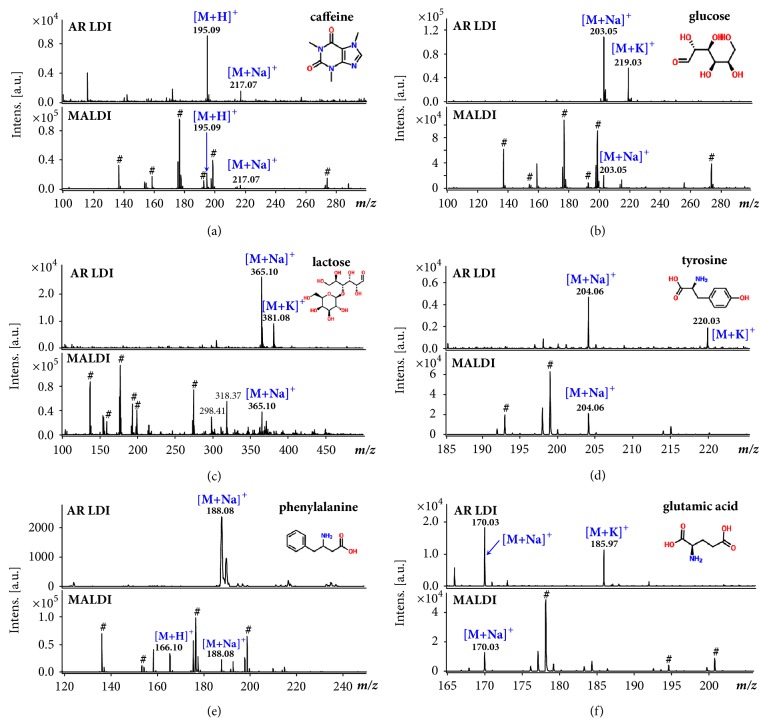
**Comparison of AR LDI with MALDI**. Mass spectra of 1 nmol (a) caffeine, (b) glucose, (c) lactose, (d) tyrosine, (e) phenylalanine, and (f) glutamic acid using AR LDI (top) and MALDI with DHB (bottom). Peaks marked with # are assigned to the clusters of DHB.

We have carried out a series of experiments to test the performance of the AR surface for analysis of a variety of molecules, with a comparison to traditional MALDI.  Figure 3 shows the mass spectra of caffeine (MW194.19), glucose (MW 180.16), lactose (MW 342.30), tyrosine (MW 181.19), phenylalanine (MW 165.19) and glutamic acid (MW 147.13) using matrix-free LDI with AR surfaces and MALDI with DHB as the matrix. The LDI sample spots were made by directly depositing 1* μ*L of sample solution (each at 1* m*M) on the AR surfaces and letting it dry in air. In MALDI experiments, the analyte and matrix were mixed in equal volumes and 2* μ*L of the mixture was spotted on the stainless-steel target for analysis.

In general, the MALDI matrix compounds possess extremely strong ionization capacity and consequently add chemical noises in low-mass ranges, as shown in Figure 3 with intense matrix clusters observed. The matrix peaks include [(DHB-H_2_0) +H]^+^ at* m/z* 137.02, [DHB+H]^+^ at* m/z* 155.03, [DHB+Na]^+^ at* m/z* 177.03, [DHB+K]^+^ at* m/z* 193.01, [DHB+2Na-H]^+^ at* m/z* 199.01, and 2 (DHB-H_2_O) +H]^+^ at* m/z* 273.08. In comparison with the conventional MALDI-MS spectra obtained with DHB, the matrix-free LDI using AR surface as substrate produced relatively lower absolute intensities of analytes but much lower interference in the low* m/z* range; thus, the peaks of the analyte ions are prominent. For most analytes detected through LDI, the major form of ions was Na^+^ adducts due to the extensive presence of Na^+^ in the metal surface, except for some molecules with high proton affinities such as caffeine, which generated [M+H]^+^ peak dominantly. The analysis of saccharides using MALDI exhibited a relatively low ionization efficiency. In contrast, when using AR LDI, the ionization process of monosaccharide and disaccharide occurred with a much higher efficiency and signal-to-noise ratio.

For analysis of common amino acids of relatively small molecular weights, the superiority of LDI using AR substrate was that the peaks could be identified much more easily without the interference. Although some slight background noises existed in the low-mass range, the analysis results above indicate that the AR substrate performs well for LDI of different types of analytes. After a thorough ultrasonic cleaning with methanol and water, the background peaks were significantly reduced. Use of AR surface as LDI substrate could provide good sensitivity for MS analysis of different kinds of molecules, with significantly reduced spectral interference. Moreover, the solvent molecules adsorbed in the microstructures via surface hydroxyl groups could potentially aid the desorption process of analyte and the ion-molecule reactions in the evaporated plume [25, 41].

### 2.4. Analysis of Mixtures

The AR surface has been successfully applied to the LDI analysis of several molecules, including neutral carbohydrates, amino acids and drug compounds. There is little or no fragmentation observed for small labile compounds (Supporting Information, [Sec supplementary-material-1]). Typically, neutral carbohydrates have a relatively low ionization efficiency, due to strong hydrogen bonding and the lack of groups for ionization through protonation. It has been established that carbohydrates have a much higher affinity for alkali metal ions than protons, therefore they would tend to be ionized in the form of Na^+^ or K^+^ adducts [42, 43]. MALDI-MS is one of the mostly used techniques for carbohydrate analysis. The advantage of MALDI resides in generating ions from carbohydrates in their underivatized forms, while other ionization methods requiring complicated derivatization procedures.

**Figure 4:**
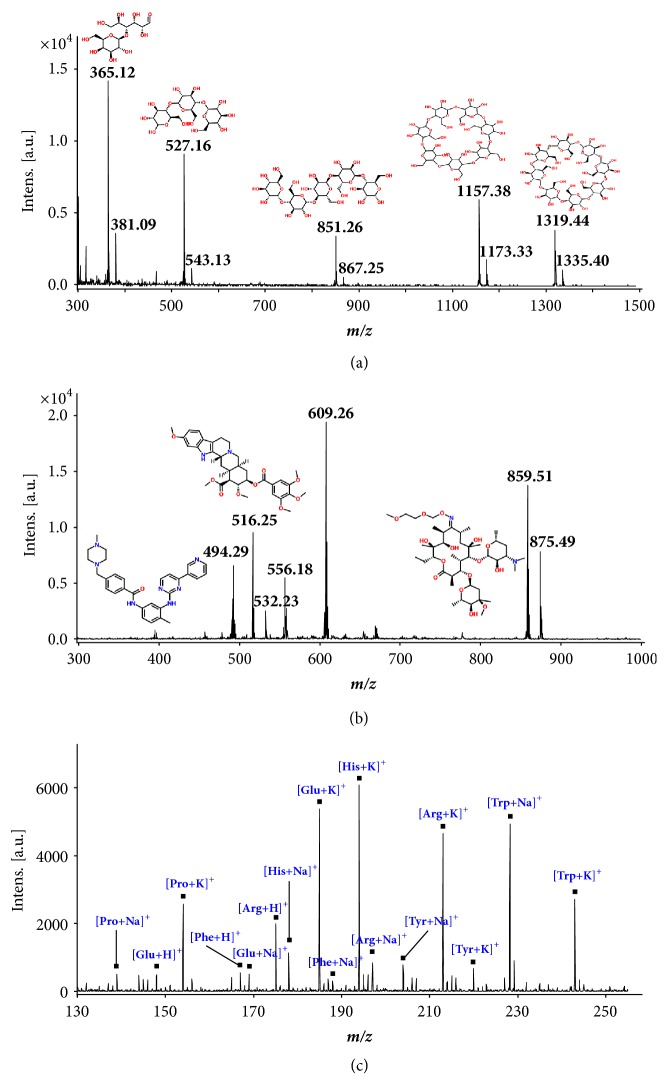
**AR LDI mass spectra** of (a) a mixture of carbohydrates (400 pmol each), (b) a mixture of drugs (400 pmol each), and (c) a mixture of amino acids (400 pmol each). The peaks in (a) at* m/z* 365.12, 381.09, 527.16, 543.13, 851.26, 867.25, 1157.38, 1173.33, 1319.44, and 1335.40 correspond to [lactose+Na]^+^, [lactose+K]^+^, [maltotriose+Na]^+^, [maltotriose+K]^+^, [maltopentaose+Na]^+^, [maltopentaose+K]^+^, [*β*-cyclodextrin+Na]^+^, [*β*-cyclodextrin+K]^+^, [*γ*-cyclodextrin+Na]^+^, and [*γ*-cyclodextrin+K]^+^, respectively. The peaks in (b) at* m/z* 494.29, 516.25, 532.25, 609.26, 631.27, 859.51, and 875.49 correspond to [imatinb+H]^+^, [imatinb+Na]^+^, [imatinib+K]^+^, [reserpine+H]^+^, [reserpine+Na]^+^, [roxithromycin+Na]^+^, and [roxithromycin+K]^+^, respectively. The amino acids in (c) are denoted by the following abbreviations: Pro: proline; Glu: glutamic acid; Phe: phenylalanine; Arg: arginine; His: histidine; Tyr: tyrosine; and Trp: tryptophan.

The AR surfaces were tested for LDI of monosaccharides, disaccharides, and other oligosaccharides of higher molecular masses. Aqueous solution 2* μ*L, containing lactose (MW 342.30), maltotriose (MW 504.44), maltopentaose (MW 828.72), *β*-cyclodextrin (MW1134.98) and *γ*-cyclodextrin (MW 1297.12), each at 1* m*M, was deposited on the AR substrate and let dry. The absolute amount of each carbohydrate was 400 pmol. The mass spectrum of the mixture is shown in Figure 4(a). The peaks at* m/z* 365.12, 527.16, 851.26, 1157.38 and 1319.44 are assigned as [lactose+Na]^+^, [maltotriose+Na]^+^, [*β*-Cyclodextrin+Na]^+^, and [*γ*-Cyclodextrin+Na]^+^, respectively.

With the AR substrates, the small carbohydrates and oligosaccharides of MW above 1000 could be efficiently desorbed and ionized without fragmentation. The Na^+^ adducts were observed as the major ion species. No [M+H]^+^ peak was observed, due to the higher affinity of the carbohydrates toward alkali metal ions than protons. The K^+^ adducts were also observed (the weak peaks on the right side of the [M+Na]^+^), with intensities much lower than the corresponding Na^+^ adducts. This was probably due to the higher concentration of Na^+^ relative to K^+^ ions present in the solvent [43]. The saccharides were at the same concentration in solution and of the absolute amount in dried sample; however, different responses were observed with LDI, with lactose of the highest ionization efficiency. The ionization efficiency for oligosaccharides of higher molecular masses was relatively lower, but the signal-to-noise ratios were still good. Effective and soft LDI of different types of carbohydrates was achieved using AR surfaces as sample substrates. A simple and effective method for ionizing neutral carbohydrates was provided, without chemical derivatization.

The LDI with AR substrate was also tested for analysis of a mixture of drug compounds, including imatinib (MW 493.60), reserpine (MW 608.68), and roxithromycin (MW 837.05). A sample solution of 2* μ*L containing these drugs, each at 1* m*M, was deposited on the AR substrate and let dry, forming a sample spot containing 400 pmol of each drug compound. The spectrum is shown in Figure 4(b). Imatinib was ionized with H^+^, Na^+^ and K^+^, reserpine predominantly with H^+^, and roxithromycin with Na^+^ and K^+^. Both analyte properties and laser fluence have an effect on the adduct formation. Using imatinib as a model compound, we observed more Na^+^ or K^+^ adducts as the laser fluence increased (Supporting Information, [Sec supplementary-material-1]). The intensities of the analyte peaks and background peaks from the substrate both increased as the laser fluence increased. Therefore, the laser fluence needs to be carefully controlled to obtain high intensities for analyte signals while minimizing the background peaks.

We have also investigated the performance of AR substrate for analysis of a mixture of amino acids. Aqueous solution of 2* μ*L containing glutamic acid (MW 147.13), histidine (MW 155.16), arginine (MW 174.20), phenylalanine (MW 165.19), proline (MW 115.13), tryptophan (MW 204.23), and tyrosine (MW 181.19), was deposited on the AR substrate and let dry, forming a sample spot with amino acids each of 400 pmol. The LDI mass spectrum is shown in Figure 4(c). All these seven amino acids could be detected in the protonated form or as adducts with Na^+^ or K^+^. Previous studies have also shown that amino acids could be cationized, dominantly by Na^+^ and K^+^ when using matrix-free LDI with different substrates [44]. Little spectral interference was observed, which made the spectra relatively simple and discernable. In a MALDI experiment for comparison, a mixture of amino acids and matrix was deposited on the stainless-steel substrate. Higher absolute intensities were observed for the analytes but abundant matrix clusters were also observed, overlapping the mass range between* m/z* 100 and 250, making the spectra very complex and undistinguished (data not shown).

**Figure 5:**
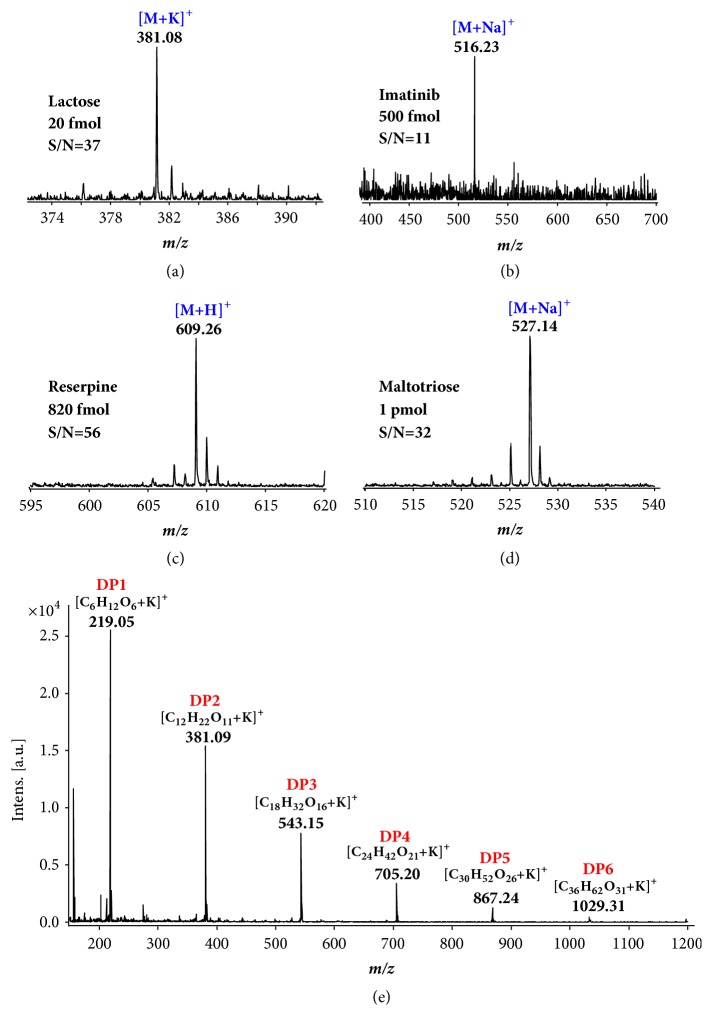
**Sensitivity evaluation for AR LDI-MS and analysis of an onion sample**. AR LDI mass spectra of (a) 20 fmol lactose; (b) 500 fmol imatinib; (c) 820 fmol reserpine and (d) 1 pmol maltotriose; and (e) saccharides from onions.

The sensitivity of LDI with AR substrate was also characterized (Figures 5(a)–5(d)). The carbohydrate samples tested were prepared with aqueous solution and the drug compounds with MeOH/H_2_O or EtOH/H_2_O solutions. Each was made at 1* m*M initially and diluted gradually to 1* n*M. Sample solution of 1* μ*L was deposited on the AR substrate and let dry to make a sample spot. The limit of detection (LOD) (determined with a S/N ≥ 3) is 2 fmol for lactose, 95 fmol for reserpine, 260 fmol for imatinib, 500 fmol for *α*-cyclodextrin, 340 fmol for maltotriose, 540 fmol for *β*-cyclodextrin, and 500 fmol for roxithromycin.

### 2.5. Direct Analysis of Plant Extracts

Direct analysis of extracts from raw samples was explored using LDI with AR substrates (Figure 5(e)). For onion extracts, rich fructans were observed. Fructan mixtures can be fairly complex. They are composed of fructosyl units and are non-reducing water-soluble carbohydrates widely existing in higher plants [45]. Lower MW fructans with degree of polymerization (DP) ranging from 2~20 are also called fructooligosaccharides, which are widely found in a variety of plants such as onions and shallots. The saccharides contained in onions are mainly glucose, sucrose and various isomeric fructooligosaccharides (DP<14) [46], such as kestose (DP=3), nystose (DP=4), and *β*-fructofuranosylnystose (DP=5) [47, 48]. Functionally, these oligosaccharides exert their crucial effectiveness in carbohydrate storage in higher plants. In addition, they play a significant role in metabolism, nutrition, and medical applications. It has been found that fructooligosaccharides can effectively promote the lipid metabolism [49], the absorption of calcium and magnesium [48], and the growth of bifidobacteria in human colon. A simple but effective direct sampling ionization technique could be very useful for analysis of complex fructan mixtures.

**Figure 6:**
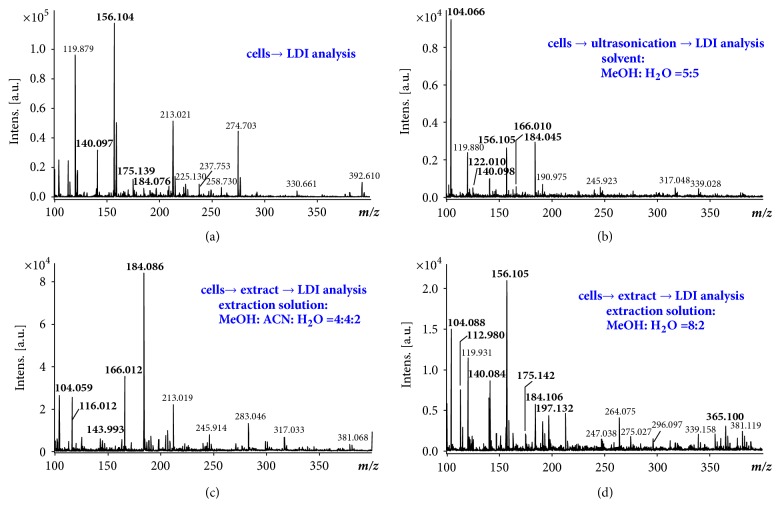
**LDI-MS analysis of* S. cerevisiae* cells.** Mass spectrum of (a) yeast cells directly deposited on the AR surface, (b) cell product after cell lysis via ultrasonication, (c) cell extract with MeOH/ACN/H_2_O (40/20/20, v/v/v), and (d) cell extract with MeOH/H_2_O (80/20, v/v).

The onion extracts were deposited on AR substrate. The LDI-MS spectrum of saccharides from onions is shown in Figure 5(e), showing saccharides of* m/z* from 200 to 1100. Based on the* m/z *values and the mass difference of 162 Da, which corresponds to the basic saccharide unit [C_6_H_12_O_6_ − H_2_O]^50^, saccharides with DP from 1 to 6 were identified. Specifically in Figure 6, the peaks at* m/z* 203.06, 219.05, 381.09, 543.15, 705.20, 867.24 and 1029.31 were assigned to the saccharide forms of [C_6_H_12_O_6_+Na]^+^, [C_6_H_12_O_6_+K]^+^, [C_12_H_22_O_11_+K]^+^, [C_18_H_32_O_16_+K]^+^, [C_24_H_42_O_21_+K]^+^, [C_30_H_52_O_26_+K]^+^, and [C_36_H_62_O_31_+K]^+^, respectively. The high concentration of K^+^ naturally contained in the onions contributed to the formation of K^+^ adducts as the dominant ion species [48]. The spectrum is very simple, with good signal-to-noise ratios and little interference. The approach of using AR surface as the LDI substrate was shown to be simple but effective, without the requirement of purification procedures.

### 2.6. Direct Analysis of Cells

Direct analysis of cells using LDI with AR substrates was also investigated. Metabolites play a significant role in many aspects of cell function, and their levels and dynamics are closely related to the cellular states [50]. Currently, MS has served as a powerful tool for metabolite analysis, due to its versatility and sensitivity [51–53]. A major challenge of using MALDI-MS toward the analysis of metabolites is the interference of matrix signals in the low-mass range, obscuring both qualitative and quantitative analysis of complex biological samples. LDI with AR substrates would certainly offer an attractive alternative solution, with no interference due to compounds from the substrate or assisting matrices.* Saccharomyces cerevisiae* cells were used as a model system for testing, which has been comprehensively studied previously using other methods [54].* S. cerevisiae* cells were treated by various combinations of solvents, with or without prior extraction, for a comparative study. The mass spectrum in Figure 6(a) was obtained by directly depositing 2~8 x 10^5^* S. cerevisiae *cells onto the AR substrate, Figure 6(b) is for the cell lysates via ultrasonication with MeOH/H_2_O (50/50, v/v) as solvent, and Figures 6(c) and 6(d) are for the cell extracts using MeOH/ACN/H_2_O (40/20/20, v/v/v) and MeOH/H_2_O (80/20, v/v) as the extracting solutions, respectively. As observed, use of different extraction solvents led to preferential detection of specific subset of metabolites. Peaks in* m/z* range for small metabolites (100~400 Da) were assigned to 13 species, including amino acids, amino acid derivatives/precursors, nucleotides, and carbohydrates, which are consistent with previous reports [52, 55, 56]. The list of the identified metabolites is summarized in Supporting Information.

The yeast metabolites were identified through accurate mass (Δ*m*/*z* ≤ 0.1) and MS/MS analysis. Databases including the KEGG database (http://www.kegg.jp/), MetaboLights (http://www.ebi.ac.uk/metabolights/), and METLIN (https://metlin.scripps.edu/) [53] were used for identity matching. Results were also compared with literature reports for confirmation [53, 54, 57, 58]. As a control experiment, the yeast cells were also analyzed via ESI-MS and ESI-MS/MS (data not shown). The Venn diagram of the species identified with different pretreatments was shown in Supporting Information, [Sec supplementary-material-1]. Five metabolite species could be identified for all samples, including 4-aminobutyric acid, uracil, 4-amino-5-hydroxymethyl-2-methylpyrimidine, histidine, and aminoadipic acid. The largest number of metabolite species was identified with cells directly deposited on the AR substrate for LDI-MS. With acetonitrile added to the extracting solvent, more amino acids could be observed, including proline and phenylalanine. Small saccharides were also observed. LDI with AR substrates was suitable for a rapid and reliable analysis of metabolites in cells.

## 3. Discussion

Previously a variety of techniques have been developed for LDI-MS analysis, achieving remarkable performance. For instance, femtomole to attomole levels of sensitivity was obtained for analyzing biomolecules in the low kDa range by DIOS [12], LOD of 5 amol for carbohydrates by carbon nanotubes or fullerene matrices [59], low picomole range for small molecules (carbohydrates, amino acids, peptides, phospholipids, and drugs) by fullerene silica substrates [60], and femtomole levels for small carbohydrates by gold nanoparticles [61]. The antireflection surfaces introduced here, however, present some unique properties attractive to biological analysis using LDI-MS. They have high light-to-heat energy conversion rate and are ideal for LDI applications. They can be easily produced through a one-step fabrication process, with highly reproducible and stable microstructures made. They are stable under normal storage conditions, highly durable, not vulnerable to cleaning process, and therefore highly reusable. In this study, we have demonstrated their potential in chemical and biological analysis using mass spectrometry. Matrix-free LDI of carbohydrates, drug compounds, and amino acids has been achieved with little chemical interferences observed, which used to be problematic for MALDI. Direct analysis of onion extracts and yeast cells indicates that the LDI with AR surfaces can be applied to investigate plant metabolism and real-world samples. The applicability of AR surfaces for LDI of larger biomolecules, such as proteins, remains to be further explored. One extraordinary advantage of the AR surfaces is that their surface properties could be easily tailored by adjusting the laser parameters and subsequent chemical modification.

## 4. Materials and Methods

### 4.1. Preparation of AR Surfaces

The AR metal surface was obtained from School of Materials Science and Engineering, Tsinghua University, China. The surfaces used in this study were fabricated on copper (99.9% purity), which was mechanically polished and cut into pieces each of a dimension 25 × 75 × 1 mm to fit into a MALDI plate (the MTP Slide Adapter II target for autoflex speed MALDI time-of-flight mass spectrometer, Bruker Daltonics GmbH, Bremen, Germany). Prior to laser processing, the surface was ultrasonically cleaned. The laser fabrication of micro/nanostructures was conducted using a TruMicro 5000 ultrafast laser system (TRUMPF GmbH + Co. KG., Ditzingen, Germany). This system generated 800 fs laser (central wavelength of 1030 nm) pulses at a repetition rate of 200 kHz [34]. The laser entered into the galvo system through beam expanding lens and multistage reflector and was focused onto the sample surface. An x–y galvo was used to scan the laser beam on the surface in a pattern of crossing lines with varied scanning intervals at atmospheric environment [34]. The main parameters used for laser fabrication included laser repetition rate* f *= 200 kHz, average power* P* of 8W, scanning interval* I* = 25 *μ*m of laser beam between two adjacent lines, diameter of the focused spot* D* = 35 *μ*m, and the scanning speed* V* = 10, 20, 40, 60, or 80 mm/s. The micro/nanostructures on Cu surfaces were characterized with a SU8010 scanning electron microscope (Hitachi, Ltd., Tokyo, Japan).

### 4.2. Materials and Chemicals

The red onion bulbs (Allium Cepa) were purchased from local market (Beijing, China). D-(+)-glucose, lactose, *α*-cyclodextrin, *β*-cyclodextrin, *γ*-cyclodextrin, and NaCl were purchased from Shanghai Aladdin Bio-Chem Technology Co.,LTD. (Shanghai, China). Maltotriose, maltopentaose, imatinib, roxithromycin, and reserpine were purchased from J&K Scientific Ltd. (Beijing, China). Caffeine, L-glutamic acid, L-histidine, L-arginine, L-phenylalanine, proline, L-tryptophan, and L-tyrosine were obtained from Beijing Biodee Biotechnology Co., Ltd. (Beijing, China). 2,5-dihydroxybenzoic acid (DHB) and other solvents including HPLC grade methanol (MeOH), acetonitrile (ACN), and ethanol (EtOH) were purchased from Sigma-Aldrich Corporation (St. Louis, Missouri, USA).

### 4.3. Sample Preparations

The carbohydrates, amino acids, and caffeine were dissolved in deionized water to make solutions at initial concentrations of 1* m*M, which subsequently were gradually diluted. Reserpine was dissolved in MeOH/H_2_O (3:7 v/v) solution. Imatinib was dissolved in MeOH/H_2_O (1:1 v/v) solution. Roxithromycin was dissolved in EtOH/H_2_O (2:8 v/v) solution. DHB solution at 20* m*g/*m*L was prepared with ACN/H_2_O (3:7 v/v). The LDI sample spots were made by directly depositing sample solution on the AR surfaces and let dry in the air. MALDI experiments were carried out for comparison. The analyte and matrix were mixed in equal volumes and the mixture was spotted on the standard steel plate and dried in the air.

### 4.4. Extraction of Oligosaccharides from Onions

Fresh onions were peeled to remove the dry outer layers, and the epidermis of onions was then chopped, grinded for 5 min, and freeze-dried. The freeze-dried onion samples (10 g) were extracted with 5* m*L deionized water for 1 h, and the sample solution was centrifuged for 20 min at 10,000 rpm to remove proteins. The supernatants were cleared by a further centrifugation step and were stored at -20°C until use for experiments [47, 48]. The extracted solutions were analyzed without any other purification or concentrating procedures.

### 4.5. Preparation of Cell Samples

The* Saccharomyces cerevisiae* was provided by School of Life Sciences, Tsinghua University. The yeast* Saccharomyces cerevisiae* BY4741 cells were cultivated in Yeast Extract Peptone Dextrose (YPD) medium, and the cells were cultured at 180 rpm and 30°C for 12 hours. The medium was created by combining 1% yeast extract, 2% peptone, and 2% glucose into a water solution [62]. The cell cultures were transferred into a centrifuge tube and centrifuged at 800 ×*g* for 10 minutes to separate the cells and medium, using Centrifuge 5810R (Eppendorf AG., Hamburg, Germany). The supernatant was discarded completely and the cell pellet was washed twice with water to thoroughly remove the medium. The cells were then stored at -80°C until use for experiments. The products of cell disruption were obtained by lysing cells in 50/50 methanol/water solutions with sonication (using Ultrasonic Homogenizer, Ningbo Scientz Biotechnology Co.,LTD., Ningbo, China). The supernatant was then pipetted out for analysis. Two samples of yeast metabolic extracts were also prepared. The metabolic activity of the yeast cells was firstly quenched using the modified procedures [63, 64]. Cells were quickly released into the center of precooled 60/40 methanol/water solution. The solution was centrifuged at -9°C for 5 min at 10000 ×*g*. The supernatant was then completely removed. Metabolites were then extracted using reported methods [65, 66]. The precooled extraction solution (80/20 methanol/water or 40/40/20 methanol/acetonitrile/water) was added to the cell pellet and the cells were resuspended; the mixture solution was put on ice for 15 minutes and then centrifuged at 4°C for 10 min at 15000 ×*g* to separate the cell debris; the supernatant was then moved to a Eppendorf tube and stored at -80°C until use for MS analysis.

### 4.6. Measurement of Contact Angle

The wetting behavior of the antireflection surfaces was evaluated by measuring the contact angle (CA) of water using OCA 15 plus video-based optical contact angle measuring device (DataPhysics Instruments GmbH, Filderstadt, Germany) [34]. Water droplets each of 4* μ*L were dispensed onto the surface and let to reach stable status. The apparent contact angle of the water droplet on each surface was measured for three times and the average value was reported. The ultrasonic treatment was performed by immersing the surfaces into a water bath in a Branson 3800 ultrasonic cleaner (Branson Ultrasonics (Shanghai) Co., Ltd., Shanghai, China) for 5 min, to achieve the surface wettability conversion.

### 4.7. MS Measurements

All the mass spectra were recorded in reflector mode using an autoflex speed MALDI time-of-flight (TOF) mass spectrometer (Bruker Daltonics GmbH, Bremen, Germany) equipped with a Nd:YAG laser at 355 nm and controlled using the Flex Control 3.3 software. The laser frequency was 2 kHz. The AR surface was installed on a modified MALDI plate. Positive ion mass spectra were acquired with an accelerating voltage of 20 kV. Each MS spectrum was obtained by averaging spectra over 500 laser shots. The attenuation ratio of the laser intensity was adjusted to balance the signal-to-noise ratio with the intensity of the signal peaks. The random walk mode was set as partial sample to improve the signal intensity and the shots at raster spot was set as 10, which meant the laser irradiation spot would automatically move every ten laser pulses.

## Supplementary Materials

Supplementary Materials The Venn diagram of the identified metabolites and tentative assignments of metabolites detected from yeast cells. The mass spectra of the same AR material for consecutive LDI-MS analyses. The AR LDI mass spectra of analytes using different laser fluences and of small labile compounds. Reflectance curves of AR surface and AR surface after LDI measurements, and the corresponding SEM images. The change in contact angle over exposure time to air.
