# Supplementary material for: Antireflection Surfaces for Biological Analysis Using Laser Desorption Ionization Mass Spectrometry
Source: Research (Wash D C). 2018 Oct 31;2018:5439729. doi: 10.1155/2018/5439729 (PMC6750120; doi:10.1155/2018/5439729)
Supplement: Supplementary Materials — The Venn diagram of the identified metabolites and tentative assignments of metabolites detected from yeast cells. The mass spectra of the same AR material for consecutive LDI-MS analyses. The AR LDI mass spectra of analytes using different laser fluences and of small labile compounds. Reflectance curves of AR surface and AR surface after LDI measurements, and the corresponding SEM images. The change in contact angle over exposure time to air. [file 5439729.f1.docx]

**Figure S1**. Reflectance curves of AR surface and AR surface after LDI measurements. The blue line corresponds to the reflectance of original AR surface, the red line corresponds to the reflectance of AR surface after ~10^3^ laser shots, and the green line corresponds to the reflectance of AR surface after ~10^6^ laser shots. The insets show the corresponding SEM images of AR surfaces.

**Figure S2**. Mass spectra for consecutive LDI-MS analyses of (A) glucose (B) caffeine and (C) lactose (1 nmol each) using the same AR material.

**Figure S3**. Mass spectra for the LDI-MS analysis of a mixture of carbohydrates (400 pmol each) using the same AR material used in Figure S2. Figure S3B is acquired after the AR surface (used in Figure S3A) has been used for 50 sample analyses. The peaks at *m/z* 203.05, 365.11, 527.16, 851.26, 995.30, 1157.36 and 1319.43 correspond to [glucose+Na]^+^, [lactose+Na]^+^, [maltotriose+Na]^+^, [maltopentaose+Na]^+^, [*α*-cyclodextrin+Na]^+^, [*β*-cyclodextrin+Na]^+^, and [*γ*-cyclodextrin+Na]^+^, respectively.

**Figure S4**. The change in contact angle over exposure time to air.

**Figure S5**. AR LDI mass spectra of 1 nmol (A) caffeine, (B) reserpine and (C) roxithromycin.

**Figure S6**. AR LDI mass spectra of imatinib using different laser fluences.

**Figure S7.** The Venn diagram of the identified metabolites. The four ellipses represent the four different cell samples, and the numbers in the ellipses represent species identified.

**Table S1.** Tentative assignments of metabolites detected from yeast cells *(S. cerevisiae)* by AR-LDI-MS.

| **Assigned**  **metabolite** | **Formula** | **Adduct** | **Calculated *m/z*** | **Measured *m/z*** | **Δ *m/z*** |
| --- | --- | --- | --- | --- | --- |
| 4-Aminobutyric acid | C_4_H_9_NO_2_ | [M+H]^+^ | 104.071 | 104.087 | -0.016 |
| Uracil | C_4_H_4_N_2_O_2_ | [M+H]^+^ | 113.035 | 112.968 | 0.067 |
| Proline | C_5_H_9_NO_2_ | [M+H]^+^ | 116.071 | 116.025 | 0.046 |
| Phenylethylamine | C_8_H_11_N | [M+H]^+^ | 122.096 | 122.013 | 0.083 |
|  |  | [M+Na]^+^ | 144.078 | 143.987 | 0.091 |
| 4-Amino-5-hydroxymethyl-2-methylpyrimidine | C_6_H_9_N_3_O | [M+H]^+^ | 140.082 | 140.120 | -0.038 |
| Glutamine | C_5_H_10_N_2_O_3_ | [M+H]^+^ | 147.076 | 146.986 | 0.090 |
| Histidine | C_6_H_9_N_3_O_2_ | [M+H]^+^ | 156.077 | 156.102 | -0.025 |
| Aminoadipic acid | C_6_H_11_NO_4_ | [M+H]^+^ | 162.076 | 162.144 | -0.068 |
|  |  | [M+Na]^+^ | 184.058 | 184.072 | -0.014 |
| phenylalanine | C_9_H_11_NO_2_ | [M+H]^+^ | 166.086 | 166.054 | 0.032 |
| Arginine | C_6_H_14_N_4_O_2_ | [M+H]^+^ | 175.119 | 175.021 | 0.098 |
|  |  | [M+Na]^+^ | 197.101 | 197.017 | 0.084 |
| Chorismic acid | C_10_H_10_O_6_ | [M+H]^+^ | 227.056 | 227.015 | 0.041 |
| Monosaccharide (sorbose, fructose, galactose, glucose，mannose) | C_6_H_12_O_6_ | [M+Na]^+^ | 203.053 | 203.018 | 0.035 |
| Disaccharide (sucrose, trehalose, melibiose, maltose) | C_12_H_22_O_11_ | [M+Na]^+^ | 365.105 | 365.074 | 0.031 |

**Figure S8**. SEM images for (A) intact AR surface and (B) AR surface after laser irradiation (~10^6^ laser shots on 1 cm^2^ substrate, energy: 50 ~100 *μ*J/pulse).
